# *Natronoglycomyces albus* gen. nov., sp. nov, a haloalkaliphilic actinobacterium from a soda solonchak soil

**DOI:** 10.1099/ijsem.0.004804

**Published:** 2021-05-17

**Authors:** Dimitry Y. Sorokin, Tatjana V. Khijniak, Alicia P. Zakharycheva, Alexander G. Elcheninov, Richard L. Hahnke, Olga V. Boueva, Elena V. Ariskina, Boyke Bunk, Ilya V. Kublanov, Lyudmila I. Evtushenko

**Affiliations:** ^1^​ Winogradsky Institute of Microbiology, Research Centre of Biotechnology, Russian Academy of Sciences, Moscow, Russia; ^2^​ Department of Biotechnology, TU Delft, The Netherlands; ^3^​ Leibniz Institute DSMZ – German Collection of Microorganisms and Cell Cultures, Braunschweig, Germany; ^4^​ All-Russian Collection of Microorganisms (VKM), G.K. Skryabin Institute of Biochemistry and Physiology of Microorganisms, Pushchino Scientific Center for Biological Research of the Russian Academy of Sciences, Pushchino, Russia

**Keywords:** *Natronoglycomyces*, * Glycomycetaceae*, soda solonchak soil, haloalkaliphilic, hydrolytic

## Abstract

A haloalkaliphilic hydrolytic actinobacterium, strain ACPA22^T^, was enriched and isolated in pure culture from saline alkaline soil (soda solonchak) in northeastern Mongolia. The isolate was facultatively alkaliphilic, growing at pH 6.5–10.5 (optimum at 7.3–9.0) and highly salt-tolerant, tolerating up to 3 M total Na^+^ as carbonates. The hydrolytic nature of ACPA22^T^ was confirmed by two different growth-dependent methods and by the presence of multiple glycosidase-encoding genes in the genome. The 16S rRNA gene-based phylogenetic analysis demonstrated that strain ACPA22^T^ formed a deep-branching lineage within the family *Glycomycetaceae,* with the highest sequence similarity value to *Glycomyces buryatensis* 18^T^ (92.1 %) and *Salininema proteolyticum* Miq-4^T^ (91.8 %). The average amino acid identity values (56.1–61.5 %) between ACPA22^T^ and other *Glycomycetaceae* members with available genomes did not exceed the threshold reported for different genera. The cell wall of ACPA22^T^ contained *meso*-diaminopimelic acid, glycine, glutamic acid and alanine in a molar ratio, characteristic of the peptidoglycan type A1γ'. The whole-cell sugars included mannose, galactose, arabinose, ribose and xylose. The major menaquinones were MK-10(Н_4_) and MK-11(Н_4_). The identified polar lipids were represented by phosphatidylethanolamine, diphosphatidylglycerol, phosphatidylglycerol, phosphatidylinositol and phosphatidylinositol mannosides. In addition, the strain had a few unidentified characteristic polar lipids, including an amine-containing phospholipid with chromatographic mobility similar to that of phosphatidylinositol. The polar lipid fatty acids were dominated by anteiso-C_17 : 0_ and iso-C_16 : 0_. The genome included a chromosome of 3.94 Mbp (G+C content 61.5 mol%) encoding 3285 proteins and two plasmids of 59.8 and 14.8 kBp. Based on the data obtained in this study, a new genus and species, *Natronoglycomyces albus* gen. nov., sp. nov, is proposed with the type strain ACPA22^T^ (=DSM 106290^T^=VKM Ac-2771^T^).

## Introduction

Specific athalassic haloalkaline ecosystems, such as soda lakes and soda soils (soda solonchaks in Russian classification [1]) with permanently high pH above 9 harbour diverse communities of haloalkaliphilic prokaryotes [2–8]. Such micro-organisms have unique adaptations allowing them to withstand high alkalinity and salinity. Furthermore, they produce various haloalkalistable metabolites and enzymes of biotechnological interest, relevant to various bioconversion and bioremediations [9, 10].

In our recent survey of soda solonchak soils from different geographical regions, including Central Asia, Caucasus, Africa and North America, a large proportion of mycelium-forming haloalkaliphilic actinobacteria with various hydrolytic activities have been isolated [8]. The isolates were mostly associated with the genera *Streptomyces* and *Nocardiopsis *, but some were distant from the genera with validly published names. This paper presents the results of taxonomic study of strain ACPA22^T^ assigned to a new genus and species of the family *Glycomycetaceae* (order *Glycomycetales *) [11–15], which currently includes five genera, namely *Glycomyces *, *Stackebrandtia *, *Haloglycomyces *, *Salininema* and *Salilacibacter* [15–21]. Several species of the above genera originate from high-salt habitats and/or show resistance to saline and alkaline conditions [19–27].

## Enrichment and isolation

The source of isolation of strain ACPA22^T^ was soda solonchak topsoil (0–5 cm deep) collected in northeastern Mongolia (Choibalsan Province: 48.06° N 113.46° E). The 1 : 5 soil:water extract had a pH of 10.6. The soluble carbonate alkalinity was 0.51 M and the total soluble salt concentration was 101 mg g^−1^. The strain was enriched and isolated into a pure culture in a sodium carbonate-buffered medium containing 0.6 M total Na^+^ and at pH 10, with carboxymethyl cellulose (CMC; 1 g l^−1^) and yeast extract (0.2 g l^−1^) as substrates [8].

## Morphology and chemotaxonomy

Growth and morphological characteristics were studied in cultures grown for 7–21 days at 28 °C on: (i) soluble starch/yeast extract medium (SS-YE; pH 10 and 0.6 M total Na^+^), (ii) the aforementioned sodium carbonate medium containing starch instead of CMC (the same pH and total Na^+^), as well as on the following agar media mixed (1 : 1 v/v) with a sodium carbonate buffer (15 g l^−1^ Na_2_CO_3_, 20 g l^−1^ NaHCO_3_, 3 g l^−1^ NaCl, 1 g l^−1^ K_2_HPO_4_, pH 9.5): International *Streptomyces* Project (ISP) 2 [27], Reasoner's 2A (R2A) [28, 29] and PYG medium (5.0 g peptone, 3.0 g yeast extract, 5.0 g glucose, 0.2 g K_2_HPO_4_). Morphological characteristics were also observed in younger (40–72 h) cultures grown at 28 °C in liquid PYG medium based on the above sodium carbonate buffer. The study was performed with light microscopy (Zeiss Axioplan Imaging 2) and scanning electron microscopy (jeol).

**Fig. 1.**
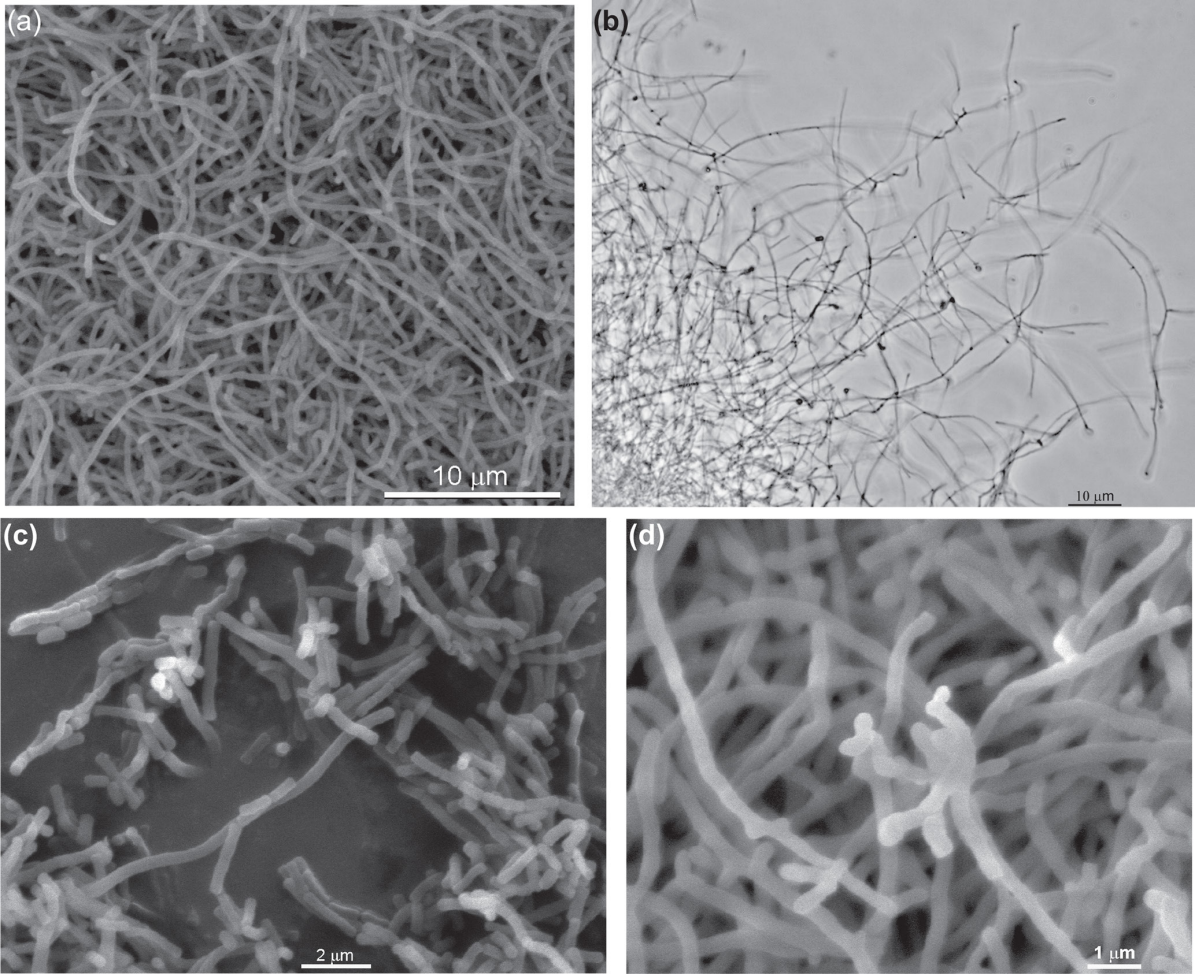
Morphology of strain ACPA22^T^ (**a, c** and **d,** scanning electron microscopy; **b,** light microscopy). (**a**) Young culture (40 h) from liquid PYG medium supplemented with CSC, pH 9. (**b**) Spore-like bodies on aerial mycelium in 7-day-old culture grown on solid starch/yeast extract medium at pH 10 and 0.6 M total Na^+^. (**c)** and** (d**) Rod-shaped fragments and spore-like bodies in 8-day-old culture grown on solid ISP 2 medium mixed with sodium carbonate buffer (see the text for details).

While growing on solid SS-YE medium at pH 10, strain ACPA22^T^ formed colonies mostly consisting of white to slightly yellowish aerial mycelium, usually straight in young cultures and moderately branched in older ones (Fig. 1). The vegetative mycelium was very weak and often seemed completely absent. On ISP 2, R2A and PYG media mixed with sodium carbonate buffer, the strain produced well developed yellowish-white to waxy vegetative mycelium and white to yellowish aerial mycelium (0.2–0.3 µm in diameter). The moderately branched vegetative mycelium mostly developed on the agar surface and did not penetrate the agar media. Both the primary and aerial hyphae fragmented into rod-shaped and square-ended elements. Short chains of two or more spherical or elongated conidia with smooth surfaces were produced on the vegetative and/or aerial hyphae by basipetal septation (fragmentation) and by acropetal budding (like that reported for some *Pseudonocardia* species) [30]. No soluble pigments were observed on any of the media tested.

Biomass for chemotaxonomic characterization of the novel strain (except for analysis of fatty acids) was obtained after growth in the PYG medium mixed (1 : 1 v/v) with a sodium carbonate buffer at 28 °C on a rotary shaker and harvested in the logarithmic growth phase (20 h). For analyses of peptidoglycan amino acids, the purified cell-wall fraction was prepared by extraction of freeze-dried cells with 0.1 N NaOH [31] followed with hydrolysis with 6M HCl at 110 °C for 20 h. Quantitative analysis of amino acids was performed by anion-exchange chromatography [32] using an automatic analyser LC200 (Biotronik) with the anion-exchange resin column loaded with the DC6a resin (Durrum). The isomer of diaminopimelic acid (DAP) was determined by thin-layer chromatography (TLC) [33]. The whole-cell sugar composition was determined after hydrolysis of cells (3 M trifluoroacetic acid, 100 °C, 6 h) on an automatic analyser LC200 using the column with anion-exchange DA-x8-11 resin (Durrum) and the dye reagent 2,2′-bicinchoninate [34]. Isoprenoid quinones were extracted from wet cells, purified according to Collins and Jones [35], and analysed with an LCQ Advantage MAX mass spectrometer (Thermo Finnigan). Polar lipids were extracted from freeze-dried cells and separated by two dimensional TLC using HPTLC silica gel 60 with a chloroform–methanol–water (65 : 25 : 4) system in the first direction and chloroform–acetic acid–methanol–water (80 : 15 : 12 : 4) in the second direction [36]. The following spray reagents were used for detection of polar lipids: 5 % ethanolic solution of phosphomolybdenum acid for all lipids; 0.25 % solution of ninhydrin in acetone for nitrogen, including the primary and secondary amines (phosphatidylethanolamine etc.); molybdenum blue spray reagent for phospholipids; α-naphthol for glycolipids; and Dragendorf reagent for choline [36, 37]. Each identified spot was also compared with respective spots on chromatograms of related reference strains for which the polar lipids were determined previously. For cellular fatty acid analysis, the cells were grown in liquid SS-SY medium until the end of the exponential growth phase (5 days) at 30 °C. Fatty acids were extracted, saponified, methylated, purified, and the methyl esters were analysed by a Trace GC Ultra coupled to a DSQ II single-quadrupole mass spectrometer (Thermo Scientific) [38] and identified by using the NIST 17 mass spectral library (https://chemdata.nist.gov/dokuwiki/doku.php?id=chemdata:nist17).

The obtained chemotaxonomic characteristics of strain ACPA22^T^ were typical of members of the family *Glycomycetaceae* [13, 16–21, 39, 40] (Table 1). The amino acids of the peptidoglycan were glycine, glutamic acid, *meso*-diaminopimelic (*meso*-DAP) and alanine in a molar ratio of 1.02 : 1.0 : 1.08 : 0.74, which corresponds to the peptidoglycan type A1γ' or A32.1 (glycine at the first position of the tetrapeptide chain) [41]. The whole-cell sugars included glucose, galactose, mannose and ribose (molar ratio, 12.1 : 2.0 : 1.0 : 5.0), with a trace amount of xylose. The respiratory quinones were represented by menaquinones MK-10(Н_4_) (~80 %), MK-11(Н_4_) (~18 %) and MK-18(Н_4_) (~2 %). The polar lipid profile included phosphatidylethanolamine, diphosphatidylglycerol, phosphatidylglycerol, phosphatidylinositol and phosphatidylinositol mannosides (Fig. S1, available in the online version of this article), which are common to many *Glycomycetaceae* species (Table 1). Along with the aforementioned lipids, ACPA22^T^ had a few other characteristic components such as unidentified amine-containing phospholipid (APL) with chromatographic mobility nearly identical to that of phosphatidylinositol, as well as unidentified glycolipids, an unidentified phosphoglycolipid and an unidentified lipid (stained only with phosphomolybdenum acid). The presence of the above amine-containing phospholipid combined with the phosphatidylinositol appears to be a marker feature of strain ACPA22^T^ (Table 1). Within the *Glycomycetaceae,* unidentified amine-containing phospholipids were reported only in three species, *Glycomyces halotolerans* [23], *Stackebrandtia albiflava* [42] and *Stackebrandtia soli* [43]. However, APL in *S. albiflava* clearly differs from APL in ASPA22^T^ by chromatographic mobility, while *Glycomyces halotolerans* and *Stackebrandtia soli* do not contain phosphatidylinositol. Another diagnostic polar lipid of strain ACPA22^T^ appears to be an unidentified phospholipid PL1 which had the chromatographic mobilitiy identical to that of phosphotidylcholine but reacted negatively to Dragendorf reagent (Fig. S1). It is worth mentioning that phosphotidylcholine was reportedly found in a few *Glycomyces* species, such as *G. artemisiae *, *G. fuscus *, *G. tarimensis* and *G. xiaoerkulensis* [22, 25, 44, 45]. The fatty acid profile of strain ACPA22^T^ included anteiso-C_17 : 0_ (56.4 %) and iso-C_16 : 0_ (14.9 %) as major components, followed by anteiso-C_17 : 1_ ω8 (7.8 %), iso-C_17 : 1 _ω8 (6.1 %), iso-C_17 : 0_ (4.6 %), iso-C_15 : 0_ (3.2 %), iso-C_16 : 1 _ω7 (1.9 %), anteiso-C_15 : 0_ (1.0 %), C_18 : 0_ (1.0 %) and C_17 : 1_ ω8 (0.8 %).

**Table 1.** Characteristics of strain ACPA22 and members of other genera in the family *Glycomycetaceae
* Strains: 1, ACPA22^T^; 2, *Haloglycomyces albus* YIM 92370^T^ [19, 21, 63]; 3, *Salilacibacter albus* J11Y309^T^ [21]; 4, *Salininema proteolyticum* Miq-4^T^ [20, 21]; 5, *Glycomyces* species [13, 40, 64]; 6, *Stackebrandtia* species [18, 27, 42, 43, 65]. *meso*-DAP, *meso*-diaminipimelic acid; PE, phosphatidylethanolamine; PG, phosphatidylglycerol; DPG, diphosphatidylglycerol; PI, phosphatidylinositol; PME, phosphatidylmethylethanolamine; APL, unknown aminophospholipids; AL, unknown aminoipid. Characteristics inherent in some representatives of the genus are indicated in square brackets.

Characteristic	1	2	3	4	5	6
Diagnostic cell-wall amino acid	*meso*-DAP, glycine	*meso*-DAP*	*meso*-DAP*	*meso*-DAP*	*meso*-DAP, glycine†	*meso*-DAP*
Whole-cell sugars	Glucose, ribose, galactose, mannose, xylose	Ribose, xylose, glucose	Glucose, ribose, xylose	Glucose, ribose, xylose	Galactose, glucose, mannose, ribose, arabinose and хylose in different combinations	Galactose, glucose, mannose, rhamnose, ribose and xylose in different combinations
Major menaquinones (more than 10%)‡	MK-10(Н_4_), MK-11(Н_4_)	MK-9(Н_4_), MK-9(Н_2_), MK-8(Н_4_), MK-10(Н_4_) or MK-9(Н_4_), MK-10(Н_4_)	MK-10(Н_4_), MK-9(Н_4_)	MK-10(Н_4_), MK-10(Н_2_), MK-9(Н_4_)	MK-10(Н_2_), MK-10(Н_4_)	MK-10(Н_4_), MK-11(Н_4_), [ MK-10(Н_6_)], [MK-11(Н_6_)]
Identified polar lipids	PE, PG, DPG, PI, PIM	PE, PG, DPG, PI, PIM	PE, PG, DPG, PI, PIM	PE, PG, DPG, PI, PIM	PG, DPG, [PI], PIM, [PE], [PC]	PE, PG, DPG, PE, PME, [PI]
Unidentified amine-containing polar lipids	APL	None	None	AL	[3 APLs] (* G. halotolerans *)	[APL] (* S. albiflava* and *S. soli *)
Predominant polar lipid fatty acids	iso-C_16 : 0_, anteiso-C_17 : 0_	iso-C_16 : 0_, iso-C_17 : 0_, anteiso-C_17 : 0_	iso-C_15 : 0_, iso-C_16 : 0_, iso-C_17 : 0_, anteiso-C_17 : 0_	anteiso-C_15 : 0_, iso-C_16 : 0_, anteiso-C_17 : 0_	anteiso-C_15 : 0_, iso-C_16 : 0_, anteiso-C_17 : 0_	iso-C_15 : 0_, anteiso-C_15 : 0_, iso-C_16 : 0_, iso-C_17 : 0_, anteiso-C_17 : 0_
G+C content (mol%)§	61.0 (HPLC); 61.5 (genome)	60.4 (genome)	63.0 (HPLC)	68.2–70.0 (HPLC)	69.9–72.8 (genome)	65.6 (genome)–71.0 (HPLC)
Na^+^ range (optimum) for growth (M)	0.05–3.0 (0.1–0.2)	0.5–3.0 (1.3–2.0)	0–1.3 (0.5–0.8)	0.5–2.5 (1.1)	0.5–2.8 (0.9)*||*	0.6–1.5 (0–1.0)*||*
pH maximum (optimum) for growth*¶*	10.5 (7.5–9.0)	9(7–7.5)	9.5 (7–7.5)	11.0* (7.0–8.5) or 9.0 (8.0)	14.0* (8.0–9.0)** 13.0* (8.0)†† 12.0* (7.0–8.0)‡‡ 11.0* (7.0 or 6.0–8.0)§§	9 (7–8)|| ||

*No available data on the the presence of glycine in the cell wall peptidoglycan (diagnostic diamino acid was determined from the whole cells).

†Glycine was found in the type and other species of the genus in which the peptidoglycan amino acids were analysed [16, 17, 39].

‡Indicated differing results reported for *Haloglycomyces albus* [19, 21]. Data on *Glycomyces* are represented by characteristics of the type species, *G. harbinensis;* other species of this genus contain menaquinones with 9, 10, 11, and/or 12 isoprene units (mostly with 10 and 11 units), with varying degree of saturation.

§Data from NCBI GENOME (http://www.ncbi.nlm.nih.gov/genome/) and IMG, Integrated microbial genomes (http://img.jgi.doe.gov/cgi-bin/w/main.cgi).

||Data on *Glycomyces xiaoerkulensis* [45] and *Stackebrandtia nassauensis* [18, 27] which reportedly show the highest resistance to NaCl among species within the respective genera.

¶Data on species which reportedly show the highest pH values within the genera *Glycomyces* and *Stackebrandtia*.

**Data for *G. albus;* initial pH value of the growth medium, ISP 4 [25].

††Data for *G. xiaoerkulensis;* initial pH value of the growth medium, ISP 3 [45].

‡‡Data for *G*. *lacisalsi* determined in the culture medium with pH buffering systems [24]; (note: indicated growth maximum pH seems questionable as this value is higher than the confirmed prokaryotic maximum [60]).

§§Data for *G. halotolerans* [23], *G. tarimensis* [22] and *G. sediminimaris* [26] determined in the culture media with pH buffering systems.

|| ||Data for *S. cavernae* determined in the culture medium with pH buffering systems [27].

*These values are unrealistic, since the media used were not properly buffered. In fact it is not possible to have a proper buffer above pH 10.5 and, therefore, growth above that value can only be verified in the pH controlled chemostat conditions.

The fatty acid type and the pattern of major components in strain ACPA22^T^ were the same as reported in other members of *Glycomycetaceae,* with *Haloglycomyces albus* and *Stackebrandtia nassauensis* being the most similar to ACPA22^T^ in a high proportion (>50 %) of predominating acid, anteiso-C_17 : 0_, in some experiments [20, 21, 27].

## Phylogenetic and genomic analyses

Genomic DNA of grown cells of strain ACPA22^T^ was extracted as described previously [46]. The SMRTbell template library was prepared according to the instructions from PacificBiosciences, following the Procedure and Checklist – Preparing Multiplexed Microbial Libraries Using SMRTbell Express Template Prep Kit 2.0. Briefly, for preparation of 10 kb libraries 1 µg genomic DNA was end-repaired and ligated to barcoded adapters applying components from the SMRTbell Express Template Prep Kit 2.0 from Pacific Biosciences. Reactions were carried out according to the manufacturer's instructions. Samples were pooled according to the calculations provided by the Microbial Multiplexing Calculator. Conditions for annealing of sequencing primers and binding of polymerase to purified SMRTbell template were assessed with the calculator in SMRTlink (PacificBiosciences). SMRT cell (1/16) was sequenced on the SequelII (PacificBiosciences) resulting in 362728 aligned subreads with mean aligned read lengths of 6847 bp. Long read genome assembly was performed with the ‘Microbial Assembly’ protocol included in SMRTlink version 8 using default parameters with exception of the target genome size, which was set to 3.5 Mbp. For strain ACPA22^T^ one circular chromosome and two circular plasmids were obtained, afterwards rotated to the chromosomal/plasmid origin (*dnaA*, *parA*). Identification of replication genes was done based on blast.

The DNA G+C content directly calculated from genome sequence was 61.5 mol%, while 61.0 mol% was determined in the purified, hydrolysed and dephosphorylized genomic DNA using HPLC [47–49]. The values obtained were lower than in other species of the family *Glycomycetaceae *, except for *Haloglycomyces albus* (60.4 mol%, genome) (Table 1).

The genome encodes three almost identical 16S rRNA genes. Primary identification of strain ACPA22^T^ was performed using NCBI blast with one of the 16S rRNA gene sequences of the strain as the query. In the 16S rRNA gene-based phylogenetic analysis, all three copies of 16S rRNA gene sequence and the sequences of type strains of all *Glycomycetaceae* species with validly published names were used, with *Rubrobacter radiotolerans* P1^T^ as an outgroup. In total there were 39 entries.

The sequences were aligned using the mafft server with G-INS-i method [50]. The phylogenetic tree was reconstructed in mega7 [51] using the maximum-likelihood method and the GTR model (G+*I*, four categories) with 1000 bootstrap replications; all positions with less than 95 % site coverage were eliminated. The alternative phylogenomic analysis based on the ‘bac120’ set of conserved single copy bacterial proteins [52] was performed as described below. The protein sequences were identified and aligned with the GTDB-tk version 1.4.0 with reference data version 95 [53]. The resulted alignment was treated using the trimAl version 1.4.1 with the following parameters: -gt 1 (full gap elimination) and -cons 50 [54]. The phylogenetic analysis was performed in the RAxML version 8.2.12 [55] with the protgammailg model of amino acid substitution; local support values were 1000 rapid bootstrap replications. Phylogenetic trees were visualized using iTOL [56].

**Fig. 2.**
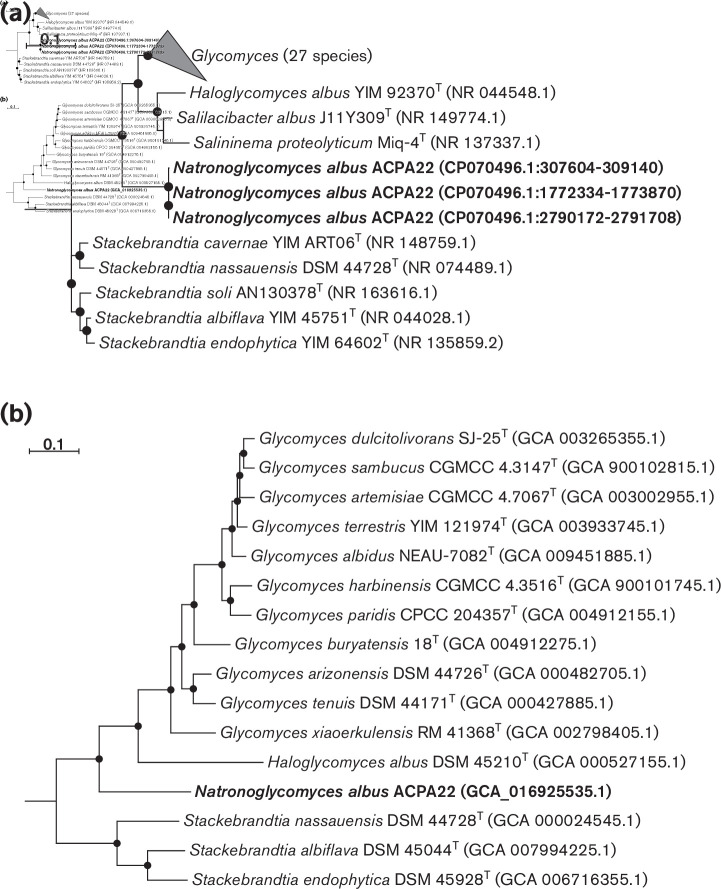
Maximum-likelihood phylogenetic trees, showing the position of strain ACPA22^T^ (in bold) within the family *Glycomycetaceae:* (**a**) the 16S rRNA gene sequence-based tree; (**b**) conserved proteins concatenated sequence-based tree. The branch lengths correspond to the number of substitutions per site with corrections associated with the models. The black circles at nodes indicate that the percentage of corresponding support values was above 50. *Rubrobacter radiotolerans* P1^T^ was used as an outgroup (not shown) in both trees.

According to the blast search, strain ACPA22^T^ showed the highest 16S rRNA gene sequence identity (~92.4 %) to *Salininema proteolyticum* IBRC-M 10908^T^ and *Glycomyces buryatensis* 18^T^. In the *Glycomycetaceae* tree based on 16S rRNA sequences, strain ACPA22^T^ formed a distinct genus-level clade (Fig. 2a), which is consistent with the distant position of this strain in the ‘bac120’-based phylogenomic tree (Fig. 2b).

The genome-based indices, average nucleotide identity (ANI) and average amino acid identity (AAI), were determined using the pyani module version 0.2.8 [57] and an AAI matrix calculator [58], respectively. The AAI values between ACPA22^T^ and other members of *Glycomycetaceae* with available genome sequences ranged from 56.1 to 61.5 % (Table S1a), which is below 65 %, a threshold proposed for different genera [59]. The ANI values (72.2–73.6 %) also did not exceed the highest value (74.1 %) calculated between members of separate genera of this family (Table S1b).

## Growth physiology

**Fig. 3.**
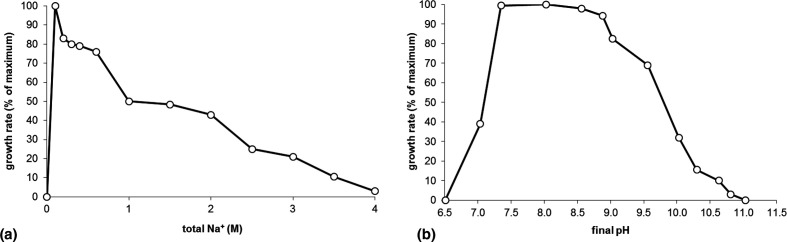
Influence of pH at 0.6 M total Na^+^ (**a**) and Na^+^ (as carbonates) at pH 10, (**b**) on growth of ACPA22^T^ on starch/yeast extract medium. The data are mean results from duplicate cultures.

The influence of temperature on growth was measured at intervals of 5 °C in the range of 5–60 °C, using SS-YE medium at pH 10 and 0.6 M Na^+^. The strain grew at 20–43 °C, with an optimum at 25–28 °C. Growth at different pH values and Na^+^ concentrations was assessed on the same medium at 0.6 M total Na+ and at pH 10, respectively. Growth was observed within the range of 0.1–3.0 M total Na^+^ (as carbonates), with an optimum at 0.1–0.2 M (Fig. 3a). For the pH profiling, the following buffer systems were used: potassium phosphate–HEPES for pH 5–8; HEPES–NaHCO_3_ for pH 8.0–8.5; and sodium carbonate–bicarbonate for pH 9–11 (the step was 0.5 pH units). Strain ACPA22^T^ grew at pH 7.0–10.5 with a broad optimum of pH 7.5–9.0, thus being a facultative alkaliphile (Fig. 3b). Both the pH growth optimum and maximum in ACPA22^T^ exceeded those of the *Glycomycetales* members. (Table 1). It is worth noting here that the pH profiling in most cases reported to date did not conform to the necessary stringency (i.e. usage of strong buffering systems compatible with biology and continuous monitoring of the pH changes during growth) [60]. Therefore, the published data need to be considered with care: for example, the pH maxima of pH 13 and 14 reported for *Glycomyces albus* [25] and *Glycomyces xiaoerkulensis* [45] are the initial pH values of the culture media that were not based on the buffering systems. On the other hand, the maximum of pH 12 for *Glycomyces lacisali* determined in the culture medium with the buffering system [24] also seems questionable because the absolute maximum pH for bacterial growth (at controlled conditions) is pH 11.3, and there is no known buffering systems for pH above 11 that would allow confident batch cultivation without continuous pH adjustment [60]. These two examples are, in fact, obviously misleading, upsetting the carefully obtained reliable knowledge on the biological borders for the high pH life.

The ability to use different sugars, sugar alcohols and organic acids as the carbon and energy sources was tested in the basal mineral medium (pH 10, 0.6 M total Na^+^) supplemented with carbon source at final concentration of 1 g l^−1^. Nitrogen from ammonium or amino acids (but not nitrate or urea) can be used by the strain, as tested with starch as the carbon and energy source. Furthermore, the isolate can also grow on peptone and yeast extract without carbohydrates. Since the organism has been enriched within the frame of search for polyhydrolytic haloalkaliphiles [8], special attention was paid to characterize its full hydrolytic potential using the two following approaches. First, the cells were inoculated in the above-mentioned basic mineral medium supplemented with 1 g l^−1^ of various polymers as a single carbon and energy source. After incubation on a rotary shaker at 150 r.p.m. and 30 °C for up to 3 weeks, growth was assessed by analysing the cell protein. Secondly, activity tests were performed in microvolume incubations with dye-conjugated polymers [61], either in the presence of rich soluble carbon/energy source in marine broth at pH 10 (6 g l^−1^ carbon source mix) or with a reduced amount of carbon sources (0.5 g l^−1^ peptone and 0.5 g l^−1^ yeast extract mix in DSMZ medium 371 supplemented with different azurin-cross-linked-polysaccharides or proteins, casein and gelatin at 25 °C for up to 14 days). Each 200 µl well of a microplate was filled with a small portion of one of the AZO-CL-polysaccharides, AZO-CL-casein (Megazym), Students–pigment–pectin and Students–pigment–gelatin and 100 µl medium. Each well was inoculated with 100 µl of a washed with fresh medium culture or 100 µl of the same medium as the control. The polymer hydrolysis results in the visible dye release. The cultivation-based and activity measurement-based approaches showed coherent, but slightly variable results, summarized in Table 2, Fig. S2. Finally, the ACPA22^T^ genome search using the dbCAN2 server [62] identified a set of genes encoding glycosidases of the families GH13 (amylase, pullulanase), GH16 (β-1,3/1,4-glucanases) and GH18, GH19 and GH20 (chitin hydrolysis) (Table 2). However, the growth test with amorphous chitin was negative. Overall, ACPA22^T^ used a wide range of water-soluble carbon sources and exhibited strong hydrolytic activity, which might be explored further for production of haloalkalistable hydrolases.

**Table 2.** Polymer-degrading potential of the soda soil actinomycete strain ACPA22^T^ in growth experiments (G), activity tests with dye-conjugated polymers (A) and encoded glucosidases in the genome (GH)

Polymer	G	A	Genomic	
			GH family	Function
**α-Glucans**				
Amylose, dextrin, cyclodextrin, pullulan glycogen	+	+	2 × GH15	Trehalase
			GH13_3	Pullulanase
			GH13_32	α-Amylase
Dextran (* Leuconostoc *)	−	−	GH49 (−)	Dextranase
**β-Glucans**				
Carboxymethyl-cellulose	−	Weak^*^	GH16_3	β-1,3(4)(6)-Endoglucanases
Amorphous cellulose	−	nd		
Barley glucan	−	−		
Laminarin	+	nd		
Lichenan	+	−		
Pachyman/curdlan	−	−	GH81 (−)	β-1,3-Endoglucanases
Xylan	−	−	GH10 (−)	β-1,4-Endoxylanase
Amorphous chitin	−	nd	GH18/19/20	Chitinases
**Other glucans including heteropolysaccharides**				
Alginate	−	nd	PL (−)	Alginate lyase
Rhamnogalacturonan	nd	Weak	GH88/105 (−)	Rhamnogalacturonyl hydrolase
β-Mannan	−	−	GH5/22/26 (−)	β-Mannanase
Arabinan	−	−	GH43	Arabinase
Galactan	−	−	GH54 (−)	β-1,4-Endogalactanase
Glucomannan	Weak	−		
Xyloglucan	−	−	GH5/GH9 (−)	Endoxyloglucanase
Arabinoxylan	−	−	GH10/GH43 (−)	Arabinase/xylanase
Pectin	−	−	PL1 (−)	Pectate lyase
Inulin	+	nd	GH32 (−)	Inulinase
**Proteins**				
Casein	+	+	nd	
Gelatin	+	+	nd	

nd, Not determined.

*Tested in plate assay with Congo red flooding.

**Negative in the dye-conjugated assay, but positive in plate assay with Lugol solution flooding.

(-), indicate absence of the corresponding GH genes; shading, Indicate positive results.

ACPA22^T^ had strong positive reactions in the catalase and oxidase tests performed with 3 % H_2_O_2_ and tetramethyl-*p*-phenylendiamine HСl, respectively. No antimicrobial activity was revealed against *Bacillus subtilis* and *Pseudomonas* species using he disk-plate technique.

Taken together, the data obtained in this study indicate that strain ACPA22^T^ should be classified as a new genus and species, for which the name *Natronoglycomyces albus* gen. nov., sp. nov. is proposed.

## Description of *Natronoglycomyces* gen. nov.

*Natronoglycomyces* (Na.tro.no.gly.co.my'ces. Gr. n. *natron*, arbitrarily derived from the Arabic n. *natrun* or *natron*, soda; N.L. masc. n. *Glycomyces* a bacterial genus name; N.L. masc. n. *Natronoglycomyces* a soda-loving *Glycomyces *-like bacterium).

Aerobic, Gram-positive and filamentous actinomycete forming branching vegetative and aerial mycelia which fragment into rod-shaped elements. Short chains of two or more spherical or elongated spores can be produced on the vegetative and/or aerial hyphae by basipetal septation (fragmentation) and by acropetal budding. The cell-wall peptidoglycan contains *meso*-diaminopimelic acid, glycine, glutamic acid and alanine in a molar ratio characteristic of the peptidoglycan type A1γ'. The major respiratory menaquinone is MK-10(Н_4_); other menaquinones may occur in lesser or minor amounts. The phospholipid profile is composed of phosphatidylethanolamine, phosphatidylglycerol, diphosphatidylglycerol, phosphatidylinositol, phosphatidylinositol mannosides and some unidentified characteristic polar lipids, including amine-containing phospholipids. The fatty acid profile is dominated by anteiso-C_17 : 0_ and iso-C_16 : 0_. A member of the family *Glycomycetaceae *, order *Glycomycetales *. The type species is *Natronoglycomyces albus*.

## Description of *Natronoglycomyces albus* sp. nov.

*Natronoglycomyces albus* (al'bus. L. masc. adj. *albus*, white, describing the colour of aerial mycelium)

White to yellowish aerial mycelium (approx. 0.2–0.3 µm in diameter) is usually well developed on agar media and branched; colonies lacking aerial hyphae may be produced on some media rich in organics. The vegetative mycelium is yellowish-white to waxy on most agar media (pH 9.5), including modified ISP 2 and R2A, based on a sodium carbonate buffer; the mycelium mostly develops on the agar surface and does not penetrate into the agar. No diffusible pigments are produced. Both the primary and aerial hyphae fragment into rod-shaped and square-ended elements. Short chains of two or more spherical or elongated spores with smooth surfaces are produced on the vegetative and/or aerial hyphae by basipetal septation (fragmentation) and by acropetal budding. The cell-wall sugars are glucose, ribose, galactose, mannose and xylose (trace amounts). The major menaquinones are MK-10(Н_4_) and MK-11(Н_4_), with a minor amount of MK-8(Н_4_). The polar lipid pattern contains phosphatidylethanolamine, diphosphatidylglycerol, phosphatidylglycerol, phosphatidylinositol, phosphatidylinositol mannosides, as well as several unidentified characteristic polar lipids, including amine-containing phospholipid with chromatographic mobilitiy similar to phosphatidylinositol and a phospholipid with chromatographic mobilitiy almost identical to that of phosphotidylcholine. The major fatty acids (>5 %) include anteiso-C_17 : 0_, iso-C_16 : 0_, anteiso-C_17 : 1_ ω8 and iso-C_17 : 1 _ω8. Salt-tolerant, with a range of total Na^+^ for growth from 0.1 to 3.0 M (optimum at 0.1–0.3 M) and facultatively alkaliphilic, with a pH range for growth from pH 7.0 to 10.5 (optimum at pH 7.5–9.0). At pH 10, the growth temperature range is 20–43 °C (optimum at 25 °C). The type strain of the species is able to use peptone, yeast extract, glucose, galactose, rhamnose, arabinose, maltose, sucrose, trehalose, melezitose, raffinose and lactose as the carbon source, but not mannose, sorbose, xylose, ribose, mannitol or inositol, as well as glucuronic and galacturonic acids. The strain hydrolyses and utilizes α-glucans for growth, including amylose, dextrin, glycogen, cyclodextrin and pullulan, as well as some β-glucans. The following polysaccharides are hydrolysed: amylose, pullulan, glycogen, rhamnogalacturonan, pachyman, laminarin and lichenan. Casein and gelatin are hydrolysed, but not olive oil. Ammonium is utilized as the nitrogen source. No antimicrobial activity against *Bacillus subtilis* and *Pseudomonas* species. The G+C content of the DNA is 61.5 mol% (genome). The type strain is ACPA22^T^ (=DSM 106290^T^=VKM Ac-2771^T^), isolated from soda solonchak soil sampled in north-eastern Mongolia.

## Supplementary Data

Supplementary material 1
