# Supplementary material for: Natronoglycomyces albus gen. nov., sp. nov, a haloalkaliphilic actinobacterium from a soda solonchak soil
Source: Int J Syst Evol Microbiol. 2021 May 17;71(5):004804. doi: 10.1099/ijsem.0.004804 (PMC8289206; doi:10.1099/ijsem.0.004804)
Supplement: Supplementary material 1 [file ijsem-71-4804-s001.pdf]

Natronoglycomyces alba **gen. nov., sp. nov., haloalkaliphilic actinobacteria from a soda solonchak soil**

Dimitry Y. Sorokin[a,b*], Tatiana V. Khijniak[a], Alicia P. Zaharycheva[a], Alexander G. Elcheninov[a], Olga V. Boueva[c], Elena V. Ariskina[c], Richard L. Hahnke[d], Boyke Bunk[d], Peter Schuman[d], Ilya V. Kublanov[a] & Lyudmila I. Evtushenko[c]

Supplementary data file

Table S1. Pairwise genome AAI (**a**) and ANI (**b**) values (in percent) between *Natronoglycomyces albus* ACPA22T (in bold) and other representatives of the *Glycomycetales* order.

Fig. S1. Polar lipid profile of strain ACPA22^T after separation by two dimensional TLC. PG, phosphatidylglycerol; DPG, diphosphatidylglycerol; PE, phosphatidylethanolamine; PI, phosphatidylinositol; PIM, phosphatidylinositol mannosides; PME, phosphatidylmonomethylethanolamine; PMI, phosphatidylinositolmannoside; PL, unknown phospholipids; APL unknown aminophospholipids; GL, unknown glicolipid; L, unknown lipid. The plates were developed with molybdatophosphoric acid.

Fig. S2. Hydrolytic activity in ACPA22 tested for polysaccharides and peptides. Cells pellets were washed with fresh medium and an aliquot of 100 µL of the culture was incubated in either 100 µL DSM medium 2216 at pH 10 or DSM medium 371 with reduced carbon sources, supplemented with the indicated polysaccharide or peptide for 14 days. AZO, azurin cross-linked polysaccharide; ink, pectin or gelatin mixed with 5% students pigment. Green frame, strongly positive; yellow frame, weekly positive.

Supplementary **Table S1a**. Pairwise genome AAI values (in percent) between *Natronoglycomyces albus* ACPA22T (in bold) and other representatives of the *Glycomycetales* ord

	**Nalb**	Snas	Send	Salb	Halb	Gten	Gari	Gxia	Gart	Gdul	Gter	Gpar	Gbur	Galb	Ghar	Gsam
***Natronoglycomyces albus* ACPA22**		**56.1**	**56.6**	**57.0**	**58.0**	**61.3**	**61.5**	**61.1**	**60.4**	**60.4**	**60.7**	**60.4**	**60.6**	**60.7**	**60.2**	**60.4**
GCF_000024545.1_*Stackebrandtia_nassauensis*_DSM_44728	**56.1**		63.3	64.6	54.6	56.7	56.7	56.6	56.4	56.3	56.6	56.3	56.0	56.4	56.0	56.4
GCF_006716355.1_*Stackebrandtia_endophytica*_DSM_45928	**56.6**	63.3		70.6	54.9	56.3	56.4	56.7	55.6	55.9	56.0	56.1	55.6	56.0	55.7	56.0
GCF_007994225.1_*Stackebrandtia_albiflava*_DSM_45044	**57.0**	64.6	70.6		55.0	57.2	57.3	57.2	56.4	56.6	56.7	56.5	56.4	56.8	56.2	56.6
GCF_000527155.1_*Haloglycomyces_albus*_DSM_45210	**58.0**	54.6	54.9	55.0		61.3	61.3	61.4	60.4	60.4	60.6	60.4	60.7	60.7	60.2	60.4
GCF_000427885.1_*Glycomyces_tenuis*_DSM_44171	**61.3**	56.7	56.3	57.2	61.3		83.8	75.9	73.0	73.3	73.8	73.5	74.4	73.8	73.9	73.4
GCF_000482705.1_*Glycomyces_arizonensis*_DSM_44726	**61.5**	56.7	56.4	57.3	61.3	83.8		76.0	73.7	73.3	73.9	73.4	74.5	73.9	74.0	73.5
GCF_002798405.1_*Glycomyces_xiaoerkulensis*_RM_41368	**61.1**	56.6	56.7	57.2	61.4	75.9	76.0		71.6	71.7	72.4	71.9	72.8	72.0	72.0	71.7
GCF_003002955.1_*Glycomyces_artemisiae*_CGMCC_4.7067	**60.4**	56.4	55.6	56.4	60.4	73.0	73.7	71.6		85.6	85.0	78.7	73.5	84.2	79.7	85.2
GCF_003265355.1_*Glycomyces_dulcitolivorans*_SJ-25	**60.4**	56.3	55.9	56.6	60.4	73.3	73.3	71.7	85.6		86.9	79.1	73.8	85.1	80.1	87.4
GCF_003933745.1_*Glycomyces_terrestris*_YIM_121974	**60.7**	56.6	56.0	56.7	60.6	73.8	73.9	72.4	85.0	86.9		79.1	73.8	86.1	79.9	86.4
GCF_004912155.1_*Glycomyces_paridis*_CPCC_204357	**60.4**	56.3	56.1	56.5	60.4	73.5	73.4	71.9	78.7	79.1	79.1		73.2	78.7	79.9	79.0
GCF_004912275.1_*Glycomyces_buryatensis*_18	**60.6**	56.0	55.6	56.4	60.7	74.4	74.5	72.8	73.5	73.8	73.8	73.2		73.9	74.4	73.4
GCF_009451885.1_*Glycomyces_albidus*_NEAU-7082	**60.7**	56.4	56.0	56.8	60.7	73.8	73.9	72.0	84.2	85.1	86.1	78.7	73.9		79.8	84.6
GCF_900101745.1_*Glycomyces_harbinensis*_CGMCC_4.3516	**60.2**	56.0	55.7	56.2	60.2	73.9	74.0	72.0	79.7	80.1	79.9	79.9	74.4	79.8		80.0
GCF_900102815.1_*Glycomyces_sambucus*_CGMCC_4.3147	**60.4**	56.4	56.0	56.6	60.4	73.4	73.5	71.7	85.2	87.4	86.4	79.0	73.4	84.6	80.0	

Supplementary **Table S1b**. Pairwise genome ANI values (in percent) between *Natronoglycomyces albus* ACPA22T (in bold) and other representatives of the *Glycomycetales* ord

	**Nalb**	Snas	Send	Salb	Halb	Gten	Gari	Gxia	Gart	Gdul	Gter	Gpar	Gbur	Galb	Ghar	Gsam
***Natronoglycomyces albus* ACPA22**		**73.1**	**72.3**	**72.4**	**72.1**	**73.5**	**73.4**	**73.3**	**73.1**	**73.2**	**73.1**	**73.1**	**73.2**	**73.1**	**73.1**	**73.2**
GCF_000024545.1_*Stackebrandtia_nassauensis*_DSM_44728	**72.7**		75.3	75.6	71.8	74.2	74.0	74.0	73.6	73.7	73.6	73.5	73.5	73.7	73.7	73.6
GCF_006716355.1_*Stackebrandtia_endophytica*_DSM_45928	**72.2**	75.2		76.8	71.6	73.1	73.0	73.1	72.7	72.9	72.8	72.9	72.8	72.8	72.9	72.8
GCF_007994225.1_*Stackebrandtia_albiflava*_DSM_45044	**72.5**	75.6	76.7		71.7	73.5	73.6	73.6	73.3	73.5	73.4	73.4	73.4	73.5	73.5	73.4
GCF_000527155.1_*Haloglycomyces_albus*_DSM_45210	**72.2**	72.2	71.9	71.8		73.0	73.0	73.3	72.6	72.7	72.8	72.4	72.8	72.6	72.9	72.7
GCF_000427885.1_*Glycomyces_tenuis*_DSM_44171	**73.2**	74.0	73.0	73.5	72.7		87.5	81.5	79.7	79.8	80.1	79.9	80.4	80.2	80.3	79.9
GCF_000482705.1_*Glycomyces_arizonensis*_DSM_44726	**73.4**	74.0	73.0	73.7	72.8	87.6		81.4	80.0	80.1	80.3	80.2	80.6	80.3	80.6	80.1
GCF_002798405.1_*Glycomyces_xiaoerkulensis*_RM_41368	**73.6**	74.1	73.2	73.6	73.2	81.6	81.4		78.7	78.9	79.3	79.0	79.6	79.2	79.2	79.0
GCF_003002955.1_*Glycomyces_artemisiae*_CGMCC_4.7067	**73.0**	73.5	72.8	73.2	72.4	79.6	79.7	78.5		87.3	87.0	82.8	79.3	86.1	83.0	86.8
GCF_003265355.1_*Glycomyces_dulcitolivorans*_SJ-25	**73.1**	73.6	72.9	73.2	72.6	79.6	79.8	78.6	87.1		88.3	83.1	79.7	87.2	83.4	88.5
GCF_003933745.1_*Glycomyces_terrestris*_YIM_121974	**73.0**	73.6	72.9	73.5	72.6	80.1	80.0	79.1	87.1	88.5		83.3	79.8	87.7	83.5	88.0
GCF_004912155.1_*Glycomyces_paridis*_CPCC_204357	**73.0**	73.6	72.8	73.4	72.6	79.9	80.0	78.9	83.0	83.3	83.3		79.6	83.0	83.8	83.2
GCF_004912275.1_*Glycomyces_buryatensis*_18	**73.2**	73.4	72.7	73.1	72.7	80.5	80.5	79.5	79.3	79.8	79.7	79.6		79.8	80.3	79.5
GCF_009451885.1_*Glycomyces_albidus*_NEAU-7082	**73.1**	73.6	72.9	73.6	72.5	80.0	80.0	79.0	86.1	87.2	87.5	82.9	79.9		83.3	86.6
GCF_900101745.1_*Glycomyces_harbinensis*_CGMCC_4.3516	**72.9**	73.6	72.9	73.4	72.5	80.3	80.3	79.0	83.0	83.6	83.4	83.6	80.3	83.4		83.3
GCF_900102815.1_*Glycomyces_sambucus*_CGMCC_4.3147	**73.1**	73.7	72.9	73.3	72.5	79.9	80.0	78.9	87.0	88.7	88.1	83.1	79.6	86.8	83.4	

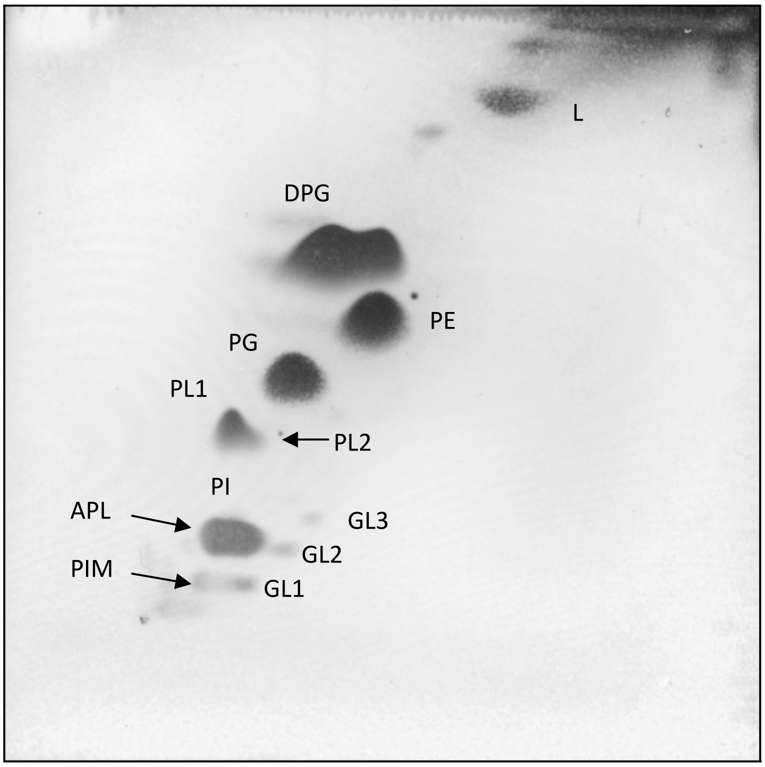

Fig. S1

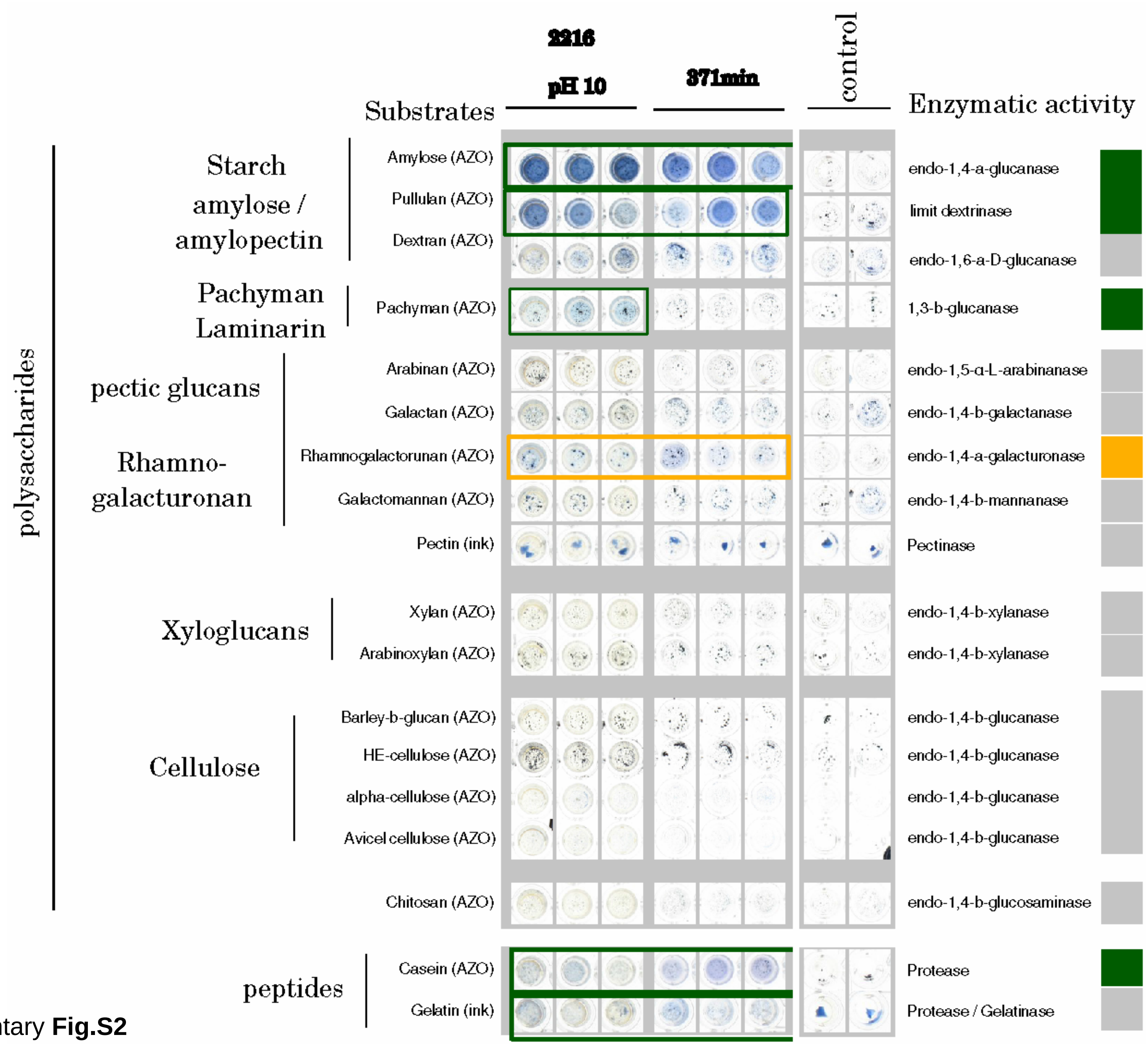

Supplementary **Fig.S2**
